# Heart Rate Variability Analysis for Seizure Detection in Neonatal Intensive Care Units

**DOI:** 10.3390/bioengineering9040165

**Published:** 2022-04-07

**Authors:** Benedetta Olmi, Claudia Manfredi, Lorenzo Frassineti, Carlo Dani, Silvia Lori, Giovanna Bertini, Cesarina Cossu, Maria Bastianelli, Simonetta Gabbanini, Antonio Lanatà

**Affiliations:** 1 Department of Information Engineering, Università degli Studi di Firenze, Via Santa Marta 3, 50139 Firenze, Italy; claudia.manfredi@unifi.it (C.M.); lorenzo.frassineti@student.unisi.it (L.F.); antonio.lanata@unifi.it (A.L.); 2 Department of Medical Biotechnologies, University of Siena, 53100 Siena, Italy; 3 Department of Neurosciences, Psychology, Drug Research and Child Health, Division of Neonatology, AOU Careggi, Largo Brambilla 3, 50134 Firenze, Italy; carlo.dani@unifi.it (C.D.); giovanna.bertini@unifi.it (G.B.); 4 Neurophysiology Unit, Neuro-Musculo-Skeletal Department, AOU Careggi University, Largo Brambilla 3, 50134 Firenze, Italy; silvia.lori@unifi.it (S.L.); cossuc@aou-careggi.toscana.it (C.C.); bastianellim@aou-careggi.toscana.it (M.B.); simonetta.gabbanini@unifi.it (S.G.)

**Keywords:** neonatal seizures, ECG, HRV, multiscale entropy, NICU

## Abstract

In Neonatal Intensive Care Units (NICUs), the early detection of neonatal seizures is of utmost importance for a timely clinical intervention. Over the years, several neonatal seizure detection systems were proposed to detect neonatal seizures automatically and speed up seizure diagnosis, most based on the EEG signal analysis. Recently, research has focused on other possible seizure markers, such as electrocardiography (ECG). This work proposes an ECG-based NSD system to investigate the usefulness of heart rate variability (HRV) analysis to detect neonatal seizures in the NICUs. HRV analysis is performed considering time-domain, frequency-domain, entropy and multiscale entropy features. The performance is evaluated on a dataset of ECG signals from 51 full-term babies, 29 seizure-free. The proposed system gives results comparable to those reported in the literature: Area Under the Receiver Operating Characteristic Curve = 62%, Sensitivity = 47%, Specificity = 67%. Moreover, the system’s performance is evaluated in a real clinical environment, inevitably affected by several artefacts. To the best of our knowledge, our study proposes for the first time a multi-feature ECG-based NSD system that also offers a comparative analysis between babies suffering from seizures and seizure-free ones.

## 1. Introduction

Neonatal epileptic seizures represent the most common clinical sign of neurological disease in newborns: the estimated incidence is about 1–5/1000 live births and 8.6/1000 in Neonatal Intensive Care Units (NICUs) [1]. They may lead to severe neurological damage, developmental delay, post-neonatal epilepsy and neonatal death in the most acute cases [2]. Therefore, the early detection of neonatal seizures is of utmost importance for an effective and timely clinical intervention [3,4]. Recently, the American Clinical Neurophysiology Society (ACNS) has defined video-electroencephalography (v-EEG) as the gold standard for the identification and quantification of neonatal seizures [5]. Indeed, the simultaneous and synchronous acquisition of EEG and video signals allows monitoring of the spontaneous electrical cerebral activity along with functional parameters, behavioral states and events causing artifacts. However, v-EEG involves a large amount of data to manage and analyze. In addition, setting up the EEG equipment and interpreting the signal requires expert staff available 24/24 h [6]. For these reasons, over the years, several studies have proposed EEG-based neonatal seizure detection systems (NSDs) to automatically detect and characterize critical events to support the clinicians’ decision in the complex diagnostic process [7]. To increase the NSD systems’ performance, researchers have focused on other possible markers of critical events. Thus, electrocardiography (ECG) has been investigated to evaluate alterations of the heart rate variability due to changes in the control of the cardiovascular system [8,9,10,11,12]. Moreover, video recordings have been examined to detect the presence of possible “unusual” movements of the newborn induced by seizure events [11,13,14,15,16,17,18,19].

Latest research findings suggest that seizures strongly influence the adults’ and newborns’ cardiocirculatory activity [12,20,21,22,23,24,25,26,27]. The normal heart rate (HR) rhythm is determined by the rate of depolarization of the sinoatrial (SA) node and is defined as the number of times per minute the heart beats. This rate is regulated by the Autonomic Nervous System (ANS), specifically by its main components, which are the parasympathetic system, which is responsible for decelerating the SA node activity and thus the HR rhythm, and the sympathetic activity that has accelerating effects on the HR. 

The term “heart rate variability” describes oscillations in consecutive cardiac cycles, and its evaluation can indicate the effect of seizures on the ANS functions [28].

The HRV signal is usually obtained from ECG and can be analyzed with different algorithms defined in time and frequency domains as well as methods derived from nonlinear dynamics. Time domain analysis uses statistical and geometrical parameters to examine HR variability. Frequency domain analysis involves estimating the power spectrum of the R-R interval time series. More specifically, the HRV power spectrum is usually divided into different spectral bands to describe the different physiological phenomena and activities of the ANS. Methods derived from nonlinear dynamics, including those based on information theory, estimate the complexity of cardiovascular dynamics [29]. Previous studies employing this methodology showed a loss of complex physiological variability under certain pathological conditions, for example, measuring the unpredictability of the R-R interval time series, and highlighted a reduced HR dynamic [30,31].

The analysis of ECG recordings is of increasing clinical interest as it could represent a reliable approach to developing helpful systems to support seizure diagnosis. The advantage is that the ECG signal is routinely performed, recordings are easier and it is less invasive and less expensive than the EEG [10,32]. Most of the computer-based systems proposed in the literature are developed to automatically detect seizure activity in adults and children. De Coomant et al. proposed a patient-independent algorithm for online epileptic seizure detection based on the analysis of single-channel ECG data from eight patients (age: 29–51 years) with temporal lobe epilepsy [33]. They used Linear Support Vector Machine and Linear Discriminant Analysis classifiers. The best performance was found for the LDA-based system, giving a Sensitivity (SEN) = 80% and Specificity (SPE) = 87%. 

Behbahani et al. proposed Multilayers Perceptron (MLP) neural networks with different numbers of hidden layers to detect seizures performing the HRV analysis on ECG data from 15 patients (9 patients with complex partial seizures, 6 with secondarily generalized seizures) (mean age 42.2 years) [34]. The MLP trained with the Levenberg–Marquardt algorithm gave SEN = 83.33%, SPE = 86.11 and Accuracy (ACC) = 84.72% for the patients with complex partial seizures, and for the patients with secondarily generalized seizures, SNE = 86.66%, SPE = 90% and ACC = 88.33%. 

Jeppesen et al. validated an HRV-based seizure detection algorithm analyzing ECG signals recorded using a wearable device [35]. They performed an offline and patient-specific analysis based on recordings from 19 patients (age: 4–62 years). The overall SEN was 56.3%. They observed that the algorithm performed better on patients with marked autonomic changes, giving an SEN = 87%.

Although these methods show appealing performance, they cannot be directly applied to newborns due to their quite different electrophysiological activity. However, some attempts to support seizure diagnosis through the ECG/HRV analysis have also been made for newborns. 

Frassineti et al. investigated the ability of the HRV analysis of discriminating between newborns with seizures and seizure-free ones in NICUs [36]. They developed a Linear SVM system that was validated on 52 full-term newborns (33 with seizures and 19 seizure-free) of a public dataset collected at the Helsinki University Hospital. The system gave an AUC = 87%.

A few other studies have focused on the HRV analysis to define efficient and reliable automatic systems able to identify neonatal seizures’ occurrence.

Greene et al. presented an ECG-based system using a Linear Discriminant (LD) classifier [9]. They considered a dataset of ECG recordings from seven full-term newborns suffering from hypoxic ischemic encephalopathy (HIE) in the NICU of the Unified Maternity Hospitals in Cork, Ireland, and the Kings’ College Hospital, London. The HRV analysis was performed extracting properties of the R-R intervals in time, frequency and information theory domains. They developed a patient-specific and a patient-independent system. The first one provided an Accuracy (ACC) = 66.04%, Sensitivity (SEN) = 75.52% and Specificity (SPE) = 57.70%, while the patient-independent system gave an ACC = 61.80%, SEN = 78% and SPE = 51.75%.

Doyle et al. investigated the usefulness of the HRV analysis to detect neonatal seizures introducing a Support Vector Machine (SVM)-based system [10]. They considered the ECG recordings of 14 full-term newborns admitted in the NICUs of the Unified Maternity Hospitals in Cork, Ireland. Concerning the HRV analysis, only a feature set defined in time and frequency domains was considered. The system results were mean Area Under the ROC Curve (AUC) = 60% and mean SEN = 60%.

However, the system performance was lower than that provided by the EEG-based systems [7,37].

Our purpose is to assess a methodological improvement to the ECG-based NSD systems. We have recently developed a Generalized Linear Model (GLM)-based system analyzing statistical HRV features and features defined in frequency and information theory domains [38]. The model was validated on 52 full-term newborns (33 with seizures and 19 seizure-free) of a public dataset collected at the Helsinki University Hospital. It gave Concatenated Area Under the ROC Curve = 69%. In this paper, we investigate the use of the SVM classifier, which is most widely used in the literature for the NSD task, as showed in [7]. We considered ECGs from a dataset of video-EEG and ECG recordings collected at the NICU of the Neuro-physiopathology Clinic, AOU Careggi, Firenze, Italy. The dataset concerns 51 full-term babies, 29 of which are control patients. To the best of our knowledge, it is the first study that compares healthy with pathological newborns, thus providing detailed and representative results from a clinical point of view. The HRV analysis is performed extracting features defined in time, frequency and information theory domains as described in [9,10]. Specifically, entropy and multiscale entropy analyses were performed, as they might provide additional cues for characterizing alterations in cardiocirculatory activity during neonatal seizures [36].

This paper is organized as follows: Section 2 describes the dataset in detail. Section 3 reports the pre-processing steps required to extract the R-R time series, the implemented methods to perform the HRV analysis, feature extraction and selection processes and the classification and validation steps. Section 4 explains the metrics employed to test the method’s performance. Section 5 resumes the achieved results. Section 6 is devoted to discussing the usefulness of HRV analysis in the NSD task. Concluding remarks are drawn in Section 7.

## 2. Dataset

A dataset of video-EEG and ECG recordings collected at the Neuro-physiopathology and Neonatology Clinical Units of AOU Careggi (Firenze, Italy) was taken into account. It was collected between March 2010 and October 2020. It consists of 51 full-term newborns with gestational age (GA) between 38 and 41 weeks. Out of 51 subjects, 29 were control patients (CP). Specifically, 20 of them did not show any seizure event, and the other 9 were healthy newborns in the Nursery. The remaining 22 newborns compose the pathological dataset as they were admitted to NICU mainly for suspected or diagnosed hypoxic-ischemic encephalopathy (HIE). More specifically, 10 out of the 22 pathological newborns showed electrographic-only seizures (EGP) characterized by abnormal changes in the EEG signal and poor clinical signs such as ocular, oral/buccal/lingual and progression movements [10,39,40,41]. The remaining 12 subjects exhibited electroclinical seizures (ECP), characterized by clinical signs coupled with EEG changes [42]. 

None of the considered newborns have heart disease that could affect the study; thus, the proposed analysis was performed on the whole dataset.

Video-EEG and ECG data were synchronously recorded for each patient using the Nemus ICU (EB Neuro S.p.A, Firenze, Italy) Galileo NT Line system, with a sampling frequency of 128 Hz. The ECG signals were pre-processed using a high-pass filter with a time constant of 0.1 s and a 50 Hz notch filter. Moreover, the one-lead ECG system was equipped with two electrodes: the active one was placed on the left axillary line at the level of the 7th rib, while the reference electrode was placed on the right clavicle.

**Figure 1:**
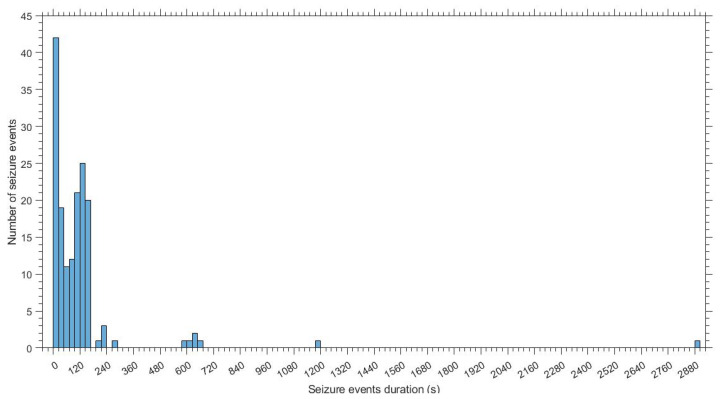
Histogram showing seizure events’ duration and seizure events’ occurrence.

The total length of recordings of the dataset was 45:46:44 h (mean duration per patient 00:53:51 h). An experienced neurologist independently annotated the seizure activity. The total seizure duration across records for the 22 pathological newborns was 03:32:11 h (EGP 00:22:07 h; ECP 03:10:04 h). The mean seizure duration per pathological patient was 00:09:39 h (EGP 00:02:13 h; ECP 00:15:50 h). The pathological dataset is reported in Table 1. Specifically, for each pathological patient, Table 1 summarizes the length of recordings, the number of seizure events and their duration and the etiology. Figure 1 shows seizure events’ duration and seizure events’ occurrence.

## 3. Methods

This section describes the methods implemented to develop an SVM-based system. SVM makes use of both the Gaussian and the Linear kernel. The system aims at recording and analyzing the ECG signal to highlight the occurrence of seizures. Moreover, when provided with a suitable user interface, it could be a valid decision support tool for the clinicians, speeding up neonatal epileptic seizure detection and thus early clinical intervention. 

The presented methods are performed on the raw ECG signals collected by the medical staff in NICUs, without artifact correction to improve the recording quality.

The proposed system is implemented in MATLAB computing environment (version 2020b [43]), OS Windows 10, 64 bit. Processor: AMD Ryzen 9 3950X 16 Core 4.7 GHz, RAM 64 Gb, GPU NVIDIA RTX 2060 Super 8 GB GDDR6.

In the following subsections, we report the processing methodology implemented into the system. It is split into three subsections. The first describes the ECG windowing process and the extraction algorithm for the interbeat interval (IBI) time series computation (also named RR time series, i.e., the time series composed by the time intervals between consecutive R peaks); the second subsection reports both the features extraction and feature selection methods. Eventually, in the third, the classification and validation steps are explained.

### 3.1. ECG Windowing and RR Time Series Extraction

The ECG signals were segmented into non-overlapping time windows, called “epochs” [44]. We considered both 60 s epochs [8,9,10] and 180 s epochs [25]. The experienced neurologist labeled the seizure events indicating both the beginning and the ending time instant of a seizure. Since an event labeled by the clinician did not precisely overlap with one or more epochs, we labeled an epoch as a “seizure epoch” if at least one sample of the signal fell inside a time interval previously classified by the experienced neurologist as a seizure event.

One of the most crucial aspects of the ECG analysis was the identification of QRS complexes for the segmentation into single beats. This ECG pre-processing enabled HRV estimation and thus found out the possible seizure’s effects on the ANS [45]. 

In particular, the localization of the R peaks was performed by implementing the Pan–Tompkins algorithm [46,47], as it was previously used for R peaks detection from neonatal ECGs [48]. Given the time instants at which R peaks were detected, we computed the time distance between each pair of consecutive R peaks obtaining the RR time series. In order to develop a fully-automatic ECG-based NSD system, no correction procedures visual-inspection-based were performed to remove false detections and ectopic beats.

### 3.2. Features Extraction

The HRV analysis was performed for each epoch, extracting a set of features defined in time, frequency and information theory domains. More specifically, we extracted 18 features for both 60 and 180 s epochs. These features were selected among the most widely used in the literature for HRV analysis [9,10,24,49]. To increase the system performance, we introduced additional features concerning the multiscale entropy, as they could provide additional useful information to characterize alterations in cardiovascular activity during neonatal seizures [36]. More specifically, we considered the Multiscale Sample Entropy (MSE) [50] and the Multiscale Distribution Entropy (MDE) [51], which were computed implementing the coarse-grained procedure [36]. The multiscale entropy indexes describe and characterize the HRV complexity at multiple time scales. Recently, the multiscale entropy analysis was successfully applied to ECG signals to detect seizures in adults [52]. We considered the Sample Entropy (SampEn) [53] one of the entropy measures most applied to physiological signals analysis. It measures the unpredictability of a time series according to Equation (1):

(1) $$\mathrm{SampEn}=-\log\frac{A}{B}$$

where A is the number of cases in which two sets of data points of length m + 1 have distance <r for a given embedding dimension m and tolerance r; B is the number of cases in which two sets of data points of length m have distance <r.

However, the SampEn is strongly dependent on the number of samples of the investigated signal, the embedding size (m) and the tolerance (r) [52]. Therefore, an erroneous and inaccurate combination of these parameters may lead to inconsistent results. To overcome this drawback, the Distribution Entropy (DistEn) was introduced [54]. It is defined in Equation (2):

(2) $$\mathrm{DistEn}\left(m\right)=-\frac{1}{\log_{2}\left(M\right)}\sum_{t=1}^{M}p_{t}\log_{2}\left(p_{t}\right)$$

where M is the number of bins of the histogram built by evaluating the distances between pairs of sets of data points of length m, $p_{t}$ is the frequency of each bin. Several studies in the literature suggested DistEn as a promising marker of arrhythmias from R-R interval time series [54,55,56].

The MSE was implemented defining the embedding size equal to 2 and the tolerance value equal to 0.2 [57]. The MDE was implemented defining the embedding size equal to 2, the time delay equal to 1 and the number of bins equal to 512. Moreover, considering the average newborn heart rate at rest (100–200 bpm [10]), we computed these features up to scale 4 for the 180 s epochs and considered only the scale 1 for the 60 s epochs. It allowed us to achieve at least 10^2^ points on each scale. This choice was made to avoid an inaccurate estimation of entropy parameters due to a coarse-grained scale at higher scales where the number of points could be too low [36,58]. All the considered features and a short description of each are reported in Table 2.

### 3.3. Feature Selection, Classification and Validation

In this study, the feature sets extracted from each epoch were normalized (zero mean and unit variance). To manage the presence of missing feature values in the dataset, an imputation process was performed. It replaces missing values with the average values of the features set. Moreover, a feature selection algorithm was applied to identify the most informative subset of features. The feature selection process allows us to reduce the number of those features that did not add significant information, making the classes separation difficult [59]. The Minimal-Redundancy-Maximal-Relevance (mRMR) algorithm was implemented. It is a filter model characterized by a low computational complexity [60]. Redundancy is defined as the average value of the mutual information between each feature and the whole set of features. The relevance is the average value of the mutual information between each feature and the target class. The mRMR algorithm maximized the mutual information between the features and the target class. It ensured that the mutual information between the new features and the already chosen ones was minimal. The features corresponding to the minimum redundancy and the maximum relevance were selected [60]. The mRMR algorithm has been tested on several clinical datasets, leading to promising performances in classification problems [61,62].

The optimal features set selection step was performed by comparing the classification performances of a Support Vector Machine (SVM) classifier fed by the full features set and then the subsets of 2 to 20 features chosen according to the order of ranking determined with mRMR [10]. 

The Linear SVM is a supervised learning technique that performs classification, finding the hyperplane that maximizes the margin between the two considered classes, thus separating data into two non-overlapping classes [63]. When data are not perfectly separable, SVM searches for the hyperplane that maximizes the margin and minimizes the misclassifications by introducing a regularization penalty term called λ. In general, the problem of maximizing the margin leads to minimizing the norm of the vector perpendicular to the hyperplane. 

This optimization problem can be solved using different routines called Solvers [64]. When data are not linearly separable, kernel functions are applied to map the samples into a high-dimensional feature space in which linear classification is possible. In that case, the Gaussian kernel approach was used.

The main hyperparameters of the Linear and the Gaussian SVMs with a short description are reported in Table 3 and Table 4, respectively [64,65]. 

Seizure detection is an inevitably unbalanced problem because a short duration usually characterizes critical events as compared to non-critical activity [66]. In our dataset, the total duration of pathological patients’ recordings was 23:37:58 h, and the total duration of their seizure activity was only 03:32:11 h. Moreover, we also considered control patients, thus making the dataset more unbalanced. The unbalanced data distribution can affect the detection and classification performance leading to biased classifier results toward the majority class to which the non-seizure data belonged. To deal with this problem, data in the non-seizure and the seizure class were managed asymmetrically by introducing the Costs C_1_ and C_2,_ respectively, thus assigning different weights to the elements of the classes during the training step [67]. 

**Figure 2:**
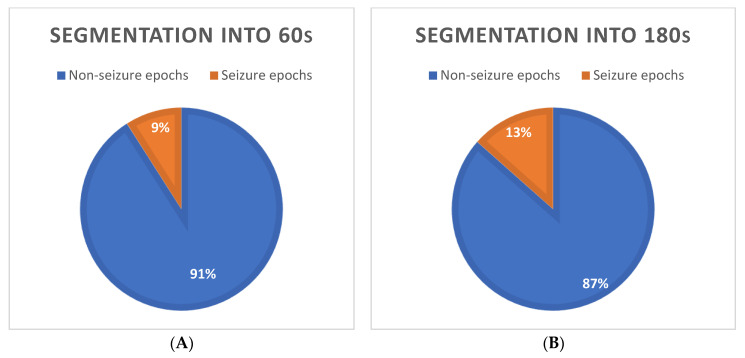
Unbalanced data in the experiments based on the segmentation of the ECG signal into the 60 s (**A**) and 180 s (**B**) epochs.

Figure 2A,B show the unbalanced data in the experiments based on the segmentation of the ECG signal into the 60 and 180 s epochs, respectively: in the first case, we had 2733 non-seizure and 277 seizure epochs; in the second one, we had 893 non-seizure and 139 seizure epochs. We labeled an epoch as a seizure if at least one sample of the signal was classified as belonging to a seizure event previously marked by the experienced neurologist.

**Table 4:** Main hyperparameters of the Gaussian SVM.

Gaussian SVM	
Hyperparameters	Short Description
Box Constraints	Regularization term that controls the number of misclassifications [68].
Kernel Scale	Scaling parameter for the input data preventing some features that have a wider range than others from becoming dominant in the kernel calculation.
Costs	Misclassification costs introduced to mitigate the class imbalance that occurs when one class has a smaller number of examples compared to the other.

To find the optimal hyperparameters values for each classifier, we performed the Grid Search optimization and the Leave One-Subject Out (LOSO) cross-validation. The Grid Search operation implemented an exhaustive search through a manually specified subset of the hyperparameter space of the learning algorithm. The LOSO method provided an almost unbiased estimation of the true generalization error. It is an iterative method: at each iteration, the training set is defined by excluding the registration of a patient, and the test set is composed of the data from that excluded patient. This process is repeated until each patient has been considered as a test subject. Thus, it performs a good evaluation of the system’s ability to generalize the classification: once trained on all the available data, it achieves performances similar to those obtained by the system with an unknown dataset [69,70]. LOSO is very useful for small datasets as no subsampling of the original dataset is performed, thus reducing the risk of overfitting. 

## 4. Performance Metrics

The main metrics used to describe the performance of seizure detection systems can be divided into epoch-based and event-based metrics [44,71]. 

The epoch-based metrics consider the segmentation of the signals into epochs. The set of analyzed epochs is divided into two classes: the seizure epochs, which are conventionally named “positive”, and the non-seizure epochs, which are named “negative”. Neonatal seizure detection can be modeled as a binary supervised problem. Generally, the classifiers developed for seizure detection provide the probability that a certain epoch belongs to the positive or negative class. System performance is obtained by evaluating the decisions made by the classifier against the manual labeling made for each epoch by the experienced neurologist.

**Figure 3:**
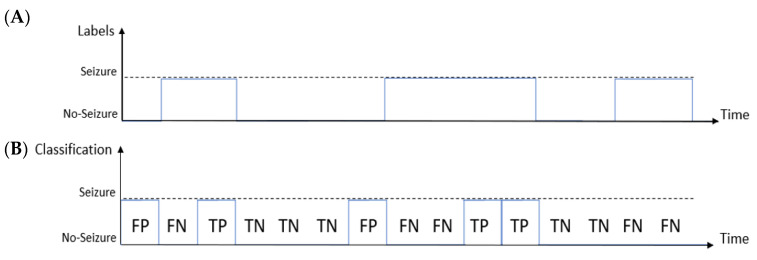
Example of the epoch-based metrics: comparison between the labeling made by the expert (**A**) and the time-windows classified by the system (**B**). In this example, the detector finds two false positive (FP), five false negative (FN), three true positive and five true negative (TN) values.

The decision made by the classifier can be represented by the so-called confusion matrix made of four categories: true positives (TP), i.e., epochs correctly labeled as seizures; false positives (FP), i.e., epochs incorrectly labeled as seizures; true negatives (TN), which refer to correctly labeled non-seizure epochs; false negatives (FN) that are epochs incorrectly labeled as non-seizure [44]. As an example, Figure 3 shows the comparison between the labeling made by the expert (Figure 3A) and the time-windows classified by the system (Figure 3B). In this example, the system correctly labeled three epochs as seizures (TP) and five epochs as non-seizures (TN), while it wrongly labeled two epochs as seizures (FP) and five epochs as non-seizures (FN).

In the literature, two main metrics are widely used to evaluate NSD systems’ performance: Sensitivity (SEN) and Specificity (SPE). SEN (Equation (3)) is defined as the ratio of the number of epochs correctly labeled as seizures and the total number of seizure epochs [44]. SPE (Equation (4)) is defined as the number of epochs correctly labeled as non-seizures over the total number of non-seizure epochs [44].

(3) $$\mathrm{SEN}=\frac{\mathrm{TP}}{\mathrm{TP}+\mathrm{FN}}$$

(4) $$\mathrm{SPE}=\frac{\mathrm{TN}}{\mathrm{TN}+\mathrm{FP}}$$

However, these metrics can be misleading evaluation measures for unbalanced problems. Thus, we also introduced the Fisher score (F1) (Equation (5)) that describes the balance between SEN and SPE.

(5) $$F1=\frac{2*\mathrm{SEN}*\mathrm{PRE}}{\mathrm{SEN}+\mathrm{PRE}}$$

where PRE stands for Precision (Equation (6)) which is defined as the ratio of the number of epochs correctly labeled as seizures and the total number of epochs labeled as seizure.

(6) $$\mathrm{PRE}=\frac{\mathrm{TP}}{\mathrm{TP}+\mathrm{FP}}$$

Most studies also report as metrics the Receiver Operator Characteristic (ROC) curves, obtained by plotting SEN against SPE (or 1-SPE), and the Area Under the ROC Curve (AUC), which is another useful parameter for comparing the performances of different systems [44]. 

**Figure 4:**
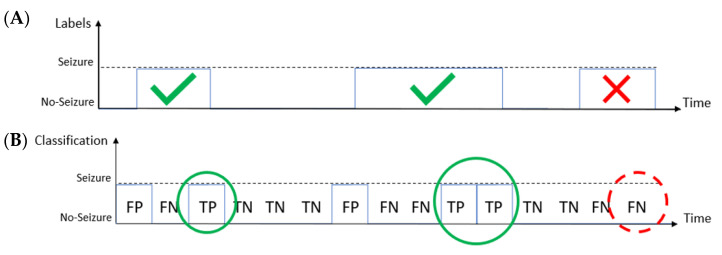
Example of the event-based metrics: comparison between the labeling made by the expert (**A**) and the time-windows classified by the system (**B**). An event is considered as correctly identified if the system detects at least one epoch during the event. In this example, the first two events are correctly identified, while the third one is not identified.

In the event-based metrics the time interval between the starting and the ending time instant of a seizure labeled by the experienced neurologist is called “event”. Thus, an event is correctly detected when the classifier correctly labels at least one seizure epoch inside the interval defined by the neurologist. Figure 4 shows an example where the classifier correctly identified three seizure epochs (solid circles in Figure 4B) during two different seizure events. Thus, it correctly detected the two seizure events (signed by the tick symbols in Figure 4A). However, the classifier did not detect a seizure epoch during the last event (dashed circle in Figure 4B), thus missing that event (signed by the cross symbol in Figure 4A).

The main event-based metrics are:

- Good Detection Rate (GDR): the overall percentage of the seizure events correctly identified by the system [44]. A seizure event is correctly identified if the system detects at least one epoch during the event.
- False Discovery Rate (FDR): the overall percentage of the seizure events incorrectly identified by the system [44].
- False Detection per Hour (FDH): the number of seizure events identified by the system in 1 h that have no overlap with the events labeled by the expert [44].

We quantified the performance of the models using all above mentioned metrics.

**Figure 5:**
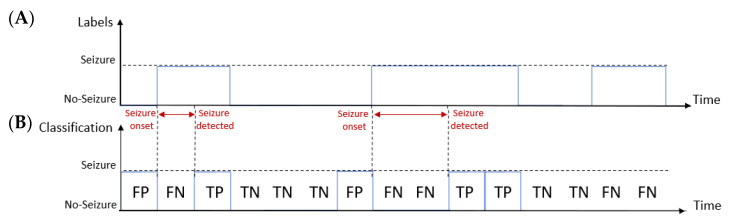
Example of the time delay metric: comparison between the labeling made by the expert (**A**) and the time-windows classified by the system (**B**). The time delay describes the time interval between the seizure detected by the algorithm and the seizure onset marked by the expert.

Additionally, we investigated the seizure detection delay by introducing the time delay metric, evaluated as the time interval between the seizure onset marked by the expert and the end of the first seizure epoch that identifies that seizure event, as shown in Figure 5.

## 5. Results

The Grid Search operation was implemented through a model for every combination of specified hyperparameters, and we evaluated each model using the above mentioned metrics. More specifically, we tested the Linear and Gaussian SVMs trained using the full set of features and the subsets selected through mRMR. We selected the models with the best average AUC values for pathological patients. Performance and hyperparameters of these models for the experiments based on the segmentation of the ECG signal into the 60 and 180 s epochs are listed in Table 5 and Table 6, respectively. 

The results reported in Table 5 and Table 6 suggest that an epoch length of 180 s is appropriate to analyze and detect seizure events in the considered dataset. Therefore, we focused on the experiment based on the signal segmented into 180 s epochs. 

**Figure 6:**
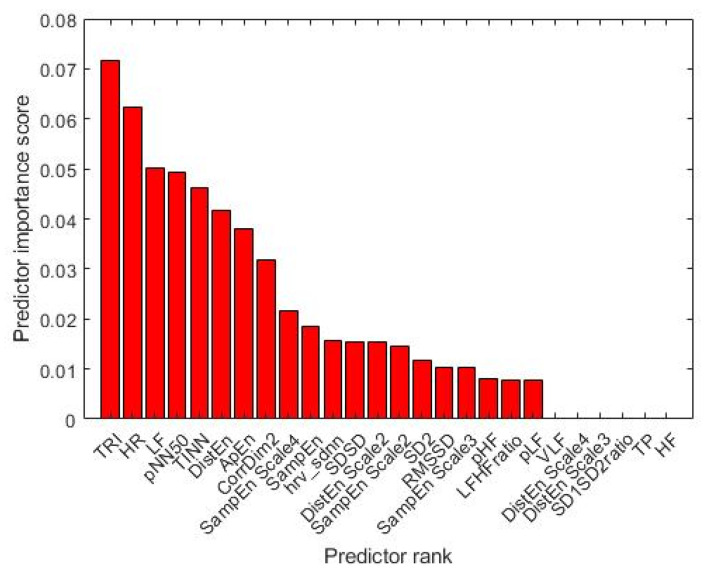
The features ranked with the mRMR algorithm for the experiment based on 180 s segmentation.

Figure 6 shows the 26 features defined for this experiment and described in Table 2. It also displays their classification relevance based on the mRMR’s predictor importance score.

**Figure 7:**
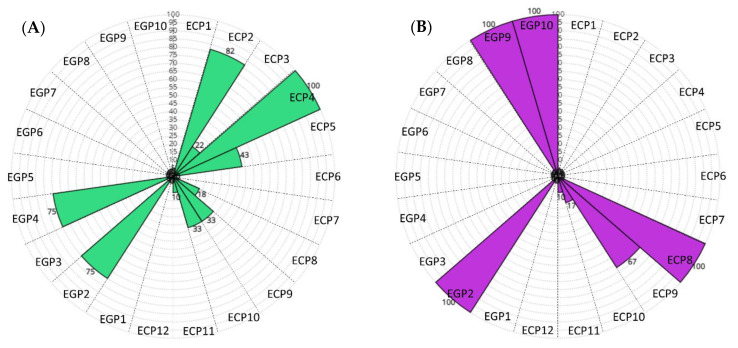
(**A**) Schematic representation of the Sensitivity values for the 22 pathological patients, iteratively obtained by the Linear SVM model trained on the full set of features during the LOSO cross-validation. A total of 10 out of 22 pathological patients are characterized by Sensitivity values >0. (**B**) Schematic representation of the Sensitivity values for the 22 pathological patients, iteratively obtained by the Linear SVM model trained on the subset of two features selected through the mRMR algorithm during the LOSO cross-validation. A total of 7 out of 22 pathological patients are characterized by Sensitivity values >0.

**Figure 8:**
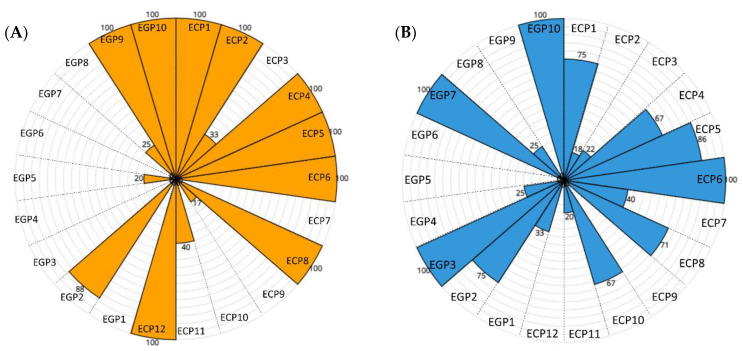
(**A**) Schematic representation of the Sensitivity values for the 22 pathological patients, iteratively obtained by the Gaussian SVM model trained on the full set of features during the LOSO cross-validation. A total of 15 out of 22 pathological patients are characterized by Sensitivity values >0. (**B**) Schematic representation of the Sensitivity values for the 22 pathological patients, iteratively obtained by the Gaussian SVM model trained on the subset of two features selected through the mRMR algorithm during the LOSO cross-validation. A total of 17 out of 22 pathological patients are characterized by Sensitivity values >0.

Moreover, we evaluated the Sensitivity values for each pathological patient (ECP = newborns with electroclinical seizures, EGP = newborns with electrographic seizures). Figure 7 shows schematic representations of the Sensitivity values for the 22 pathological patients obtained by the Linear SVM models. A circular sector describes each patient (EGP and ECP), and each Sensitivity value is represented by the radius: 0% is at the center, and 100% is at the outer circumference. More specifically, the model trained using the full set of features resulted in 10 out of 22 pathological patients characterized by Sensitivity values >0, as shown in Figure 7A. The model trained with the subset of 2 features selected through the mRMR algorithm, shown in Figure 7B, gave worse results: only 7 out of 22 pathological patients were characterized by Sensitivity values >0. Similarly, Figure 8A,B show the Sensitivity values for the pathological patients obtained by the Gaussian model trained with the full set of features and the subset of two features selected with the mRMR algorithm, respectively. The first one gave 15 patients with Sensitivity values >0. The second one gave 17 patients with Sensitivity values >0.

**Figure 9:**
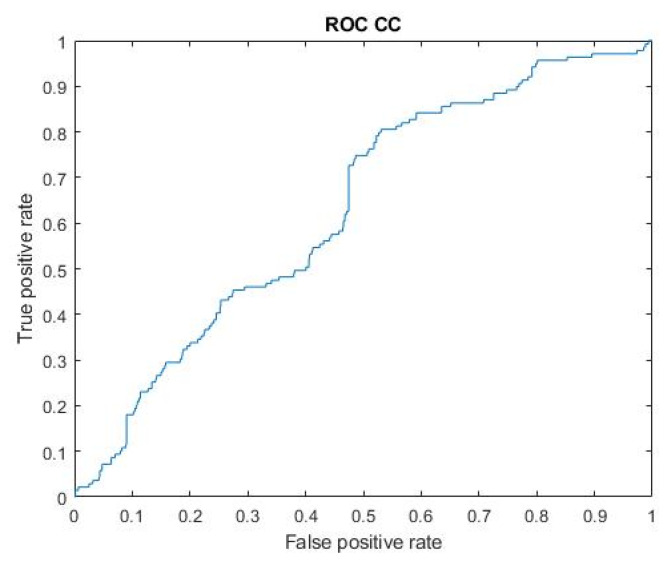
The concatenated ROC evaluated on the SVM Gaussian kernel-based system with highest AUC. All the recordings are linked together into a single recording.

Considering the overall performance, the Gaussian SVM model trained using a subset of two features seems to provide a good tradeoff between a high AUC value (mean ± standard error: 62 ± 5%) and a large number of patients with Sensitivity >0. We also calculated the concatenated AUC (AUCcc), defined as the Area Under the ROC curve, across all the concatenated recordings [72]. This ROC curve, shown in Figure 9, was built linking together all the recordings from control and pathological patients. The AUCcc was equal to 63%.

## 6. Discussion

This study aimed to develop an SVM-based system to automatically detect neonatal seizures in NICUs by investigating ECG recordings. In order to analyze the effects that seizures might have on the Autonomic Nervous System (ANS), the HRV analysis was performed based on features defined in time, frequency and information theory domains [9,10]. In addition, entropy and multiscale entropy features were also considered.

According to the main ECG-based scientific approaches for the NSD task [8,9,10,25], two experiments were performed, segmenting the ECG signals into the 60 and 180 s epochs, respectively. These choices are based on the following considerations. The Task Force of the European Society of Cardiology and the North American Society of Pacing and Electrophysiology suggested time windows of 5 min as standard time duration for the HRV analysis in adults [28]. As the average newborn heart rate at rest is higher than that of adults, a 3 min window should contain a comparable number of beats with respect to the 5 min in adults [24]. Moreover, in our dataset, 89% of seizures last less than 3 min, and about 40% of them last less than 1 min, as shown in Figure 1. Therefore, in most cases windows lasting 1 min are too short to include a seizure event. Additionally, as suggested in [73], changes in the autonomic (ANS) functions related to epilepsy might be observed in the ictal period and the interictal and postictal periods, both close to the ictal events. Thus, longer time windows might be able to catch these variations accurately.

Based on these considerations, the results reported in Table 5 and Table 6 suggest that, for our dataset, an epoch length of 180 s achieved better ability in detecting and analyzing seizure events than 60 s epochs, although it leads to an increase in the seizure detection delay, which is assessed with the time delay metric. Indeed, this measure is heavily influenced by the time duration of the epochs in which the signal is segmented and the processing time required to run the algorithms [7]. In the experiment based on the signal segmentation into 60 s epochs, the time delay ranged between (mean ± standard error) 42 ± 0.6 and 116 ± 10 s. In the experiment based on the signal segmentation into 180 s epochs, the time delay ranged between (mean ± standard error) 117 ± 13 and 141 ± 4 s. Thus, although it achieved better overall performance, as shown in Table 6, the system developed segmenting the ECG signals into 180 s epochs would not seem suitable for real-time applications. However, the use of overlapping time windows, which could reduce the system’s response time, should be investigated.

As mentioned above, we labeled an epoch ad a seizure epoch if at least one sample of the signal fell inside a time interval previously classified by the experienced neurologist as a seizure event. This choice could seem extreme, leading to reduced performance. However, finding a clinical univocal definition of neonatal seizure is still challenging, and thus also finding an operational definition for the NSD task is very tricky. Some papers consider a seizure epoch when 50% of the time window contains seizure [9]. This choice is not feasible for our dataset segmented into 180 s epochs, because about 66% of seizures last less than 90 s, and the risk of missing them would occur. According to the American Clinical Neurophysiology Society (ACNS) [5], which defined an electrographic neonatal seizure as “a sudden, abnormal EEG event, defined by a repetitive and evolving pattern with a minimum 2 μV peak-to-peak voltage and duration of at least 10 s”, we tested our methods re-labeling an epoch as a “seizure epoch” if it contains at least 10 s of seizure previously classified by the experienced neurologist as a seizure event. However, we achieved lower performance than those reported in Table 5 and Table 6. Indeed, ACNS also remarks that the choice of 10 s is conventional and arbitrary; thus, other seizure durations should also be evaluated in NSD experiments. Based on this evidence, the choice of considering at least one signal sample could be cautionary, even if drastic. The AUC values reported in Table 6 show that the Linear and Gaussian SVM models trained using a subset of two features look promising. They gave AUC values equal to (mean ± standard error) 58 ± 5 and 62 ± 5%. These models were trained using the Triangular Index (TRI) and the average heart rate measurements, as shown in Figure 6. The TRI is a geometrical measure that estimates the overall HRV [28]. Its main advantage is its relative insensitivity to artifacts [74,75]. To the best of our knowledge, no other studies analyzed the relationship between the TRI values and the seizure events in newborns. However, some works developed for adults [76] and children [77] have observed that TRI values were lower in epileptic patients than in controls, reflecting reduced parasympathetic and increased sympathetic activities during seizures [74]. This research evidence supports our results, suggesting that TRI is a robust and valuable measure for discriminating between seizure and non-seizure epochs.

The average heart rate feature is important as well. Indeed, heart rate is one of the most common autonomic manifestations of seizures in the neonatal period [40,78]. Several authors reported significant changes in heart rate during adult epileptic seizures [27,79,80,81]. These changes were also observed in neonatal seizures. Greene et al. considered five recordings from four epileptic neonates, and their study showed a significant increase in heart rate during clinical and subclinical seizures [82]. Statello et al. found that HR values were significantly higher during seizure events than the interictal periods far from the seizure events [25].

Table 6 shows high values of AUC, appealing FDH (mean ± standard error: 1 ± 0.2 h^−1^) and FDR (mean ± standard error: 4 ± 1%) values obtained by the Linear SVM model trained using the two features described above. However, it achieves a rather low average Sensitivity (mean ± standard error: 22 ± 9%), GDR (mean ± standard error: 25 ± 9%) and F1 (mean ± standard error: 13 ± 1%). Indeed, as shown in Figure 7B, it gives Sensitivity values >0 only for 7 out of 22 pathological patients. Instead, in our opinion, a reliable NSD should give a Sensitivity >0 for the largest number of pathological patients, as missed seizures could have a higher impact on the newborn’s health status than false positives. Moreover, detecting at least one seizure epoch for each pathological patient may speed up the diagnostic decision process and thus early clinical intervention. It highlights the recordings on which the medical staff should focus its attention for further analysis. Therefore, the Gaussian SVM model trained using the two features (TRI and average HR) should be preferable, although it gave worse values of FDH (mean ± standard error: 3 ± 0.3 h^−1^) and FDR (mean ± standard error: 16 ± 1%). On the other hand, it provided a high AUC value (mean ± standard error: 62 ± 5%) and increased Sensitivity (mean ± standard error: 47 ± 8%) and F1 (mean ± standard error: 29 ± 5%) values. At the same time, it gave Sensitivity values >0 for 17 out of 22 pathological patients, as shown in Figure 8B. Moreover, the Gaussian SVM model gave higher Sensitivity values for 64% of pathological patients than those given by the Linear SVM.

Concerning Sensitivity and Specificity, our results are slightly worse than those reported by Greene et al. [9] and Doyle et al. [10]. However, a comparison is challenging because a standardized performance assessment framework for the seizure detection task is currently missing, and the metrics used to report NSD systems results vary in the literature [44]. Moreover, our study was retrospective as we trained and validated our methods on ECGs collected by the medical staff in NICUs, with no artifact correction to improve the recording quality. Thus, raw signals could be affected by noisy artifacts such as those due to natural newborns’ motor activity and therapeutic maneuvers performed by the clinicians. Furthermore, most NSD systems proposed in the literature are evaluated on private datasets only. Greene et al. [9] proposed a Linear SVM for automatic HR-based seizure detection based on a dataset of eight ECG recordings from seven full-term newborns admitted in NICU for HIE. This dataset contained 101 h of recordings. Doyle et al. [10] proposed a Linear SVM that was evaluated on a dataset of 208 h of recordings from 14 full-term newborns admitted in NICU for HIE.

In this study, we considered a dataset of about 46 h of recordings from the Neuro-physiopathology NICU Clinic, AOU Careggi, Firenze, Italy. Even though our dataset is made of fewer hours of recordings, it concerns a wider set of subjects which is made of 51 full-term babies, 22 of which have seizures, and 29 are control patients. To the best of our knowledge, our study is the first one that proposes an ECG-based NSD system focusing the analysis also on a set of control newborns, thus offering a more representative picture of the performance of the system in a real clinical environment. Indeed, also considering healthy newborns would provide an effective NSD system to support clinicians in distinguishing between pathological and healthy patients.

Overall, the performances of the ECG-based systems are lower than those provided by the EEG-based systems [37]; thus, they seem not suitable for clinical implementation. EEG is the basic technique for detecting neonatal seizures as it allows recording and analyzing the spontaneous electrical cerebral activity [5,38]. The reasons behind this gap in performance between EEG-based NSD and ECG-based systems could be several and heterogeneous [7,9,10,11,36]. A detailed investigation of such reasons is out of the aim of this work, but it could be addressed in future studies. However, there is still room for improvement in HRV features for neonatal seizure detection. For example, searching for different and specific nonlinear features that can better describe the heart rate dynamics in neonatal patients could be advisable [24]. Furthermore, there is still a lack of information about possible relationships between the Autonomic Nervous System and the Central Nervous System during neonatal seizures. Thus, understanding and characterizing the interactions between the two systems [83,84,85] during or close to ictal events in the newborn might add helpful information in the seizure detection problem. Moreover, to the best of our knowledge, for ECG/HRV analysis, no deep-learning method was proposed in the literature for neonatal seizure detection. Considering the improvement obtained by DL techniques on EEG, these methods should also be evaluated on NSD experiments with ECG signals [7].

## 7. Conclusions

This study investigates the use of HRV analysis to detect neonatal seizures in the NICUs. Specifically, the HRV analysis was investigated introducing additional features to those most widely used in the literature for HRV analysis [9,10,24,49] describing multiscale entropy and exploring its capability to detect abnormal heart rate dynamics due to seizure events. We developed a Gaussian SVM-based system based on the segmentation of the ECG signal into the 60 and 180 s epochs, finding out that the epoch length of 180 s is more suitable for analyzing our dataset. 

Our findings may confirm that HRV analysis can be helpful to characterize the dynamics of ictal events in the newborn. Moreover, although at present ECG-based NSD cannot replace EEG-based NSD, when properly developed, they may represent a valid alternative to EEG systems, especially when these are not readily available.

## Figures and Tables

**Table 1:** Description of recordings of patients with electrographic (EGP) and electroclinical (ECP) seizures. Recordings length, number of seizure events and their duration and etiology for each pathological patient are reported.

Patients	Record Length (h)	Number of Seizure Events	Seizure Events Duration			Etiology
			Average	Min	Max	
EGP1	01:08:29	1	00:03:40	00:03:40	00:03:40	Metabolic
EGP2	01:09:34	8	00:00:24	00:00:12	00:00:43	HIE
EGP3	01:10:15	1	00:00:31	00:00:31	00:00:31	Other
EGP4	00:56:28	4	00:00:16	00:00:07	00:00:22	HIE
EGP5	01:02:30	3	00:01:45	00:01:18	00:02:00	HIE
EGP6	01:17:02	4	00:00:12	00:00:08	00:00:16	HIE
EGP7	01:05:53	1	00:00:50	00:00:50	00:00:50	HIE
EGP8	00:45:40	3	00:01:28	00:01:19	00:01:40	HIE
EGP9	01:20:39	1	00:00:29	00:00:29	00:00:29	Other
EGP10	00:58:22	1	00:01:40	00:01:40	00:01:40	Other
ECP1	00:48:12	1	00:48:12	00:48:12	00:48:12	Genetic
ECP2	00:54:31	13	00:01:48	00:00:27	00:09:49	Metabolic
ECP3	01:04:14	6	00:02:49	00:01:20	00:04:40	Stroke
ECP4	01:18:36	5	00:00:33	00:00:16	00:00:50	Genetic
ECP5	01:00:10	3	00:07:54	00:01:56	00:19:45	Other
ECP6	01:10:02	7	00:01:05	00:00:33	00:02:23	HIE
ECP7	00:54:06	1	00:10:26	00:10:26	00:10:26	Stroke
ECP8	01:00:43	10	00:02:53	00:01:19	00:11:04	Other
ECP9	01:30:08	5	00:00:48	00:00:22	00:01:16	HIE
ECP10	00:41:16	3	00:03:52	00:00:20	00:10:10	Stroke
ECP11	01:01:02	8	00:00:52	00:00:26	00:02:01	HIE
ECP12	01:20:06	3	00:01:58	00:01:19	00:02:31	HIE
Total	23:37:58	92				

**Table 2:** Features extracted for HRV analysis. Domain, unit of measure and a short description are provided for each feature.

	Feature	Unit of Measure	Short Description
Time domain	SDSD	(ms)	Standard deviation of successive R-R interval differences
	SDNN	(ms)	Standard deviation of R-R intervals
	RMSDD	(ms)	Root mean square of successive differences
	pNN50	(%)	Probability of R-R intervals > 50 ms e < −50 ms
	TRI	-	Area of the histogram of R-R intervals divided by its maximum height
	TINN	(ms)	Width of the R-R intervals histogram evaluated trough triangular interpolation
	CD	-	Correlation dimension
	SD2	(ms)	Standard deviation of Poincarè plot along the line-of-identity
	SD1SD2ratio	-	Ratio of standard deviation of Poincarè plot perpendicular to the line-of-identity to standard deviation of Poincarè plot along the line-of-identity
	HR	(beats/min)	Average heart rate
Frequency domain	VLF	(ms^2^)	Spectral density (computed through FFT) of the linear interpolated R-R tachogram up to 0.04 Hz (very low frequency) [25]
	LF	(ms^2^)	Spectral density (computed through FFT) of the linear interpolated R-R tachogram between 0.04 and 0.3 Hz (low frequency) [25]
	HF	(ms^2^)	Spectral density (computed through FFT) of the linear interpolated R-R tachogram between 0.3 and 1.3 Hz (high frequency) [25]
	LFHFratio	-	Ratio between spectral density of low frequency parts and high frequency parts
	TP	(ms^2^)	Total spectral density
	pLF	(%)	Percentage of spectral density of low frequency parts to total spectral density minus the spectral density of very low frequency parts
	pHF	(%)	Percentage of spectral density of high frequency parts to total spectral density minus the spectral density of very low frequency parts
Information theory domain	ApEn	-	Approximate Entropy
	Multiscale DistEn Scale (1–4)	-	Multiscale Distribution Entropy from scale 1 to scale 4 for the 180 s epochs; at scale 1 for the 60 s epochs
	Multiscale SampEn Scale (1–4)	-	Multiscale Sample Entropy from scale 1 to scale 4 for the 180 s epochs; at scale 1 for the 60 s epochs
Total	20 (60 s epochs)/26 (180 s epochs)		

**Table 3:** Main hyperparameters of the Linear SVM.

Linear SVM	
Hyperparameters	Short Description
λ	Regularization penalty term introduced to search for the hyperplane that maximizes the margin and minimizes the misclassifications.
Costs	Misclassification costs introduced to mitigate the class imbalance that occurs when one class has a smaller number of examples compared to the other.

**Table 5:** Models with the best average AUCs obtained in the experiment based on the segmentation of the ECG signal into 60 s implementing the LOSO cross-validation.

Model	N° Features	Hyperparameters	AUC (%)	SEN (%)	SPE (%)	GDR (%)	FDH (h^−1^)	FDR (%)	F1 (%)	Time Delay (s)
			(Mean ± Standard Error)							
**Linear SVM**	Full feature set (20)	λ = 10^−5^ Solver: dual C_1_ = 1; C_2_ = 2	52 ± 4	24 ± 7	89 ± 3	27 ± 8	2 ±1	4 ± 1	12 ± 4	56 ± 3.5
	Features selected through mRMR (20)	λ = 10^−8^ Solver: dual C_1_ = 1; C_2_ = 7	54 ± 3	26 ± 8	87 ± 3	36 ± 8	3 ± 0.4	5 ± 1	15 ± 4	116 ± 10
**Gaussian SVM**	Full feature set (20)	Box Constraint: 1 Kernel Scale: 5 C_1_ = 1; C_2_ = 7	52 ± 3	29 ± 8	84 ± 4	34 ± 9	4 ± 1	6 ± 1	16 ± 5	42 ± 0.6
	Features selected with mRMR (5)	Box Constraint: 0.5 Kernel Scale: 1 C_1_ = 1; C_2_ = 7	54 ± 3	24 ± 8	85 ± 2	27 ± 9	3 ± 1	6 ± 1	16 ± 5	55 ± 3

**Table 6:** Models with the best AUCs obtained in the experiment based on the segmentation of the ECG signal into 180 s implementing the LOSO cross-validation.

Model	N° Features	Hyperparameters	AUC (%)	SEN (%)	SPE (%)	GDR (%)	FDH (h^−1^)	FDR (%)	F1 (%)	Time Delay (s)
			(Mean ± Standard Error)							
**Linear SVM**	Full feature set (26)	λ = 10^−7^ Solver: dual C_1_ = 3; C_2_ = 1	56 ± 5	22 ± 7	87 ± 3	31 ± 8	1 ± 0.2	6 ± 1	20 ± 6	141 ± 4
	Features selected with mRMR (2)	λ = 10^−7^ Solver: dual C_1_ = 1; C_2_ = 40	58 ± 5	22 ± 9	77 ± 5	25 ± 9	1 ± 0.2	4 ± 1	13 ± 1	138 ± 15
**Gaussian SVM**	Full feature set (26)	Box Constraint: 0.5 Kernel Scale: 25 C_1_ = 1; C_2_ = 5	50 ± 4	51 ± 1	61 ± 5	58 ± 10	2 ± 0.3	10 ± 1	27 ± 6	117 ± 13
	Features selected through mRMR (2)	Box Constraint: 5 Kernel Scale: 0.1 C_1_ = 1; C_2_ = 200	62 ± 5	47 ± 8	67 ± 3	62 ± 9	3 ± 0.3	16 ± 1	29 ± 5	123 ± 3

## Data Availability

All data are available from the corresponding author upon reasonable requests.
